# Towards multi-omics synthetic data integration

**DOI:** 10.1093/bib/bbae213

**Published:** 2024-05-06

**Authors:** Kumar Selvarajoo, Sebastian Maurer-Stroh

**Affiliations:** Biomolecular Sequence to Function Division, BII, (A*STAR), Singapore, 138671, Republic of Singapore; Synthetic Biology Translational Research Program, Yong Loo Lin School of Medicine, NUS, Singapore, 117456, Republic of Singapore; School of Biological Sciences, Nanyang Technological University (NTU), Singapore 639798, Republic of Singapore; Biomolecular Sequence to Function Division, BII, (A*STAR), Singapore, 138671, Republic of Singapore; Synthetic Biology Translational Research Program, Yong Loo Lin School of Medicine, NUS, Singapore, 117456, Republic of Singapore

**Keywords:** synthetic data, process-driven, data-driven, machine learning, multi-omics

## Abstract

Across many scientific disciplines, the development of computational models and algorithms for generating artificial or synthetic data is gaining momentum. In biology, there is a great opportunity to explore this further as more and more big data at multi-omics level are generated recently. In this opinion, we discuss the latest trends in biological applications based on process-driven and data-driven aspects. Moving ahead, we believe these methodologies can help shape novel multi-omics-scale cellular inferences.

Since the turn of the millennium, through multi-omics technological advances, biological and medical fields have been faced with the challenge of dealing with big data generation. Without proper data analytics, these data are enigmatic. Thus, the last two decades of research have been dedicated to understanding variability, noise and bias in omics-wide data to analyze and make sense of the resulting large volume of generally high-quality data. In certain cases, the collection of such data is not simple or straightforward. For example, clinical data contain sensitive patient information, and single-cell omics data can be costly and time consuming to generate. How can we overcome or address such issues?

In the fields of big data and artificial intelligence, synthetic data have been generated and used to investigate human behaviors and pattern recognition [1]. Synthetic data are data that are mainly generated using statistical methodologies or machine learning, i.e. artificially, rather than from actual or experimental events. It is created using algorithms, like data augmentation, and is used for a wide range of applications, including as test data for new products and tools, and for model training and validation without compromising consumer privacy [2]. It has also been shown to be inexpensive, as it reduces the number and time taken for experiments, and combines well with the real data to increase the overall number of observations. Therefore, synthetic data generation has been adopted for almost three decades across a variety of research fields, with more recent applications coming into the clinical and omics fields [3].

Synthetic data generation may be classified into two major categories: process-driven and data-driven methods [4] (Figure 1). In biology, process-driven methods can generate data based on computational or mathematical models of an underlying biochemical process, such as signal transduction or metabolic pathways [5]. Examples include dynamic or kinetic models based on ordinary differential equations, stochastic models based on the Gillespie algorithm or Monte Carlo simulations and agent-based or cellular automata modeling. Here, the models are first developed to explain an observed behavior and then subsequently used to generate simulated or synthetic data using the same model for different conditions or situations. For example, in studying TRAIL signaling for cancer resistance, a dynamic model was used to predict a novel target that significantly enhances cancer cell death [6], which was subsequently tested and validated experimentally in bulk or population cells [7] (Figure 1A). This model was then used to generate 1000 single-cell dynamic data, which are not experimentally plausible due to very small expression values [8]. Thus, the *in silico* or synthetic approach emphasizes the understanding of systems-level effects of interactions between species or agents on the system as a whole, especially in areas where experiments cannot reach.

**Figure 1:**
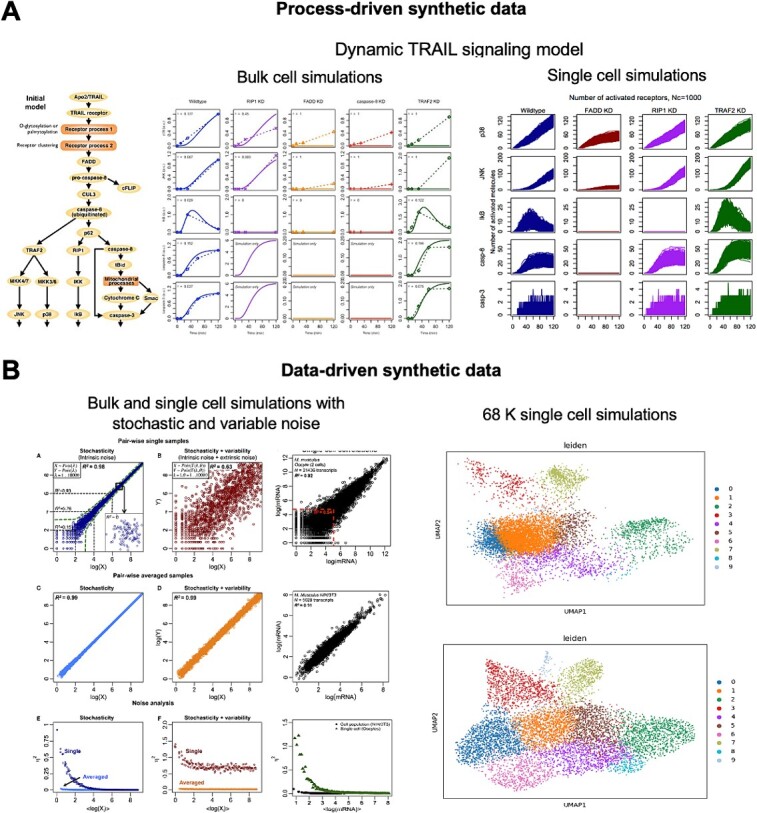
Synthetic data generation. (**A**) Process-driven. Left: for bulk cells an initial signaling model is developed using known mechanistic biochemical reactions and the corresponding experimental data for TRAIL signaling [6]. Right: the bulk validated TRAIL model is used to generate 1000 single cells data synthetically [8]. (**B**) Data-driven. Left: statistical models are used to general transcriptome-wide expression data scatter plots for bulk and single cells [10]. Right: a VAE-based model used to generate UMAP plots of 68 K single cells data, top (real) and bottom (synthetic using ACTIVA [11]).

On the other hand, data-driven methods generate synthetic data that have been trained on actual or observed data. Here, generalized linear regression models and non-linear methods, such as self-organizing maps, random forest and neural networks can be adopted. Notably, a statistical modeling procedure that learns a joint probability distribution is able to generate synthetic data fully with partial real data. More recently, deep learning methods have been used to generate two most popular types of generative AI models today: variational autoencoder (VAE) and generative adversarial network (GAN) models. These newer techniques can improve data utility by feeding models with more and more data. For biological applications, so far, synthetic data has been largely generated for single cell and spatial omics [9, 10]. For example, ACTIVA, an improved VAE model, can generate synthetic transcriptomics data utilizing data augmentation that significantly improves the classification of rare subtypes (Figure 1B, right panels) [11]. Moving forward, these models could include physics and statistical knowledge, such as using scale-free network, power-law and lognormal distributions observed in biological data, as a means to constraint false prediction and further improve the overall machine learning outcome.

Although useful, as described, it is important to note some of the key limitations of synthetic data generation. First, in real data, we often pick up outliers of interest; synthetic data will not be able to reproduce them easily as they are usually trained to pick up general patterns of the majority data. Second, the quality of synthetic data will highly correlate with the input data, thus, thorough quality checks on the original data are necessary before the machine learning process. Third, user acceptance may be challenging since it is ‘learnt’ data and not everyone might see or appreciate the benefits. Fourth, in every synthetic data generation, proper skillset, time and effort are key for training and quality check evaluation, which are pinnacle to the overall predictive quality.

Despite this, synthetic data research has gained interest and momentum recently, and in the near future can be used to generate multi-omics datasets for more integrative biological applications. Although this may result in more challenges in trusting the entire simulation, machine learning techniques can be further improved to fine-tune the comparison between multi-omics synthetic data and actual experimental data by using sophisticated feature extraction and dimension reduction methods.

**Key Points**

- Computational and machine learning models are playing key roles in biological understanding.
- Synthetic data research is relatively new in biology, and mainly focuses on single omics datasets.
- Further developments for generating multi-omics synthetic datasets for more integrative biological applications are required.

## Notes

**Kumar Selvarajoo** is a senior principal investigator at BII, heading the Computational Biology & Omics laboratory. He is also an adjunct associate professor at the NUS School of Medicine and NTU School of Biological Sciences.

**Sebastian Maurer-Stroh** is the executive director of BII and an adjunct professor at the department of biological sciences, NUS.
